# The effect of a brief mindfulness induction on processing of emotional images: an ERP study

**DOI:** 10.3389/fpsyg.2015.01391

**Published:** 2015-09-11

**Authors:** Marianna D. Eddy, Tad T. Brunyé, Sarah Tower-Richardi, Caroline R. Mahoney, Holly A. Taylor

**Affiliations:** ^1^Cognitive Science Team, U.S. Army Natick Soldier Research, Development, and Engineering CenterNatick, MA, USA; ^2^Department of Psychology, Tufts UniversityMedford, MA, USA

**Keywords:** event-related potentials, mindfulness, focused breathing, emotion

## Abstract

The ability to effectively direct one’s attention is an important aspect of regulating emotions and a component of mindfulness. Mindfulness practices have been established as effective interventions for mental and physical illness; however, the underlying neural mechanisms of mindfulness and how they relate to emotional processing have not been explored in depth. The current study used a within-subjects repeated measures design to examine if focused breathing, a brief mindfulness induction, could modulate event-related potentials (ERPs) during emotional image processing relative to a control condition. We related ERP measures of processing positive, negative, and neutral images (the P300 and late positive potential – LPP) to state and trait mindfulness measures. Overall, the brief mindfulness induction condition did not influence ERPs reflecting emotional processing; however, in the brief mindfulness induction condition, those participants who reported feeling more decentered (a subscale of the Toronto Mindfulness Scale) after viewing the images had reduced P300 responses to negative versus neutral images.

## Introduction: Mindfulness and Emotion

Our everyday lives are influenced by emotion. The ability to regulate our emotions and suppress irrelevant emotional distracters is essential for operating in an environment laden with emotionally salient events. Much research has focused on cognitive reappraisals during emotion regulation, however, practices, such as mindfulness, which alter your general state and receptiveness to incoming stimuli have not been explored extensively as methods for regulating one’s emotions. Over the past several years, mindfulness practices have grown in popularity and have been established as effective interventions for mental and physical illness (for reviews, see Baer, 2003; Greeson, 2009; Bohlmeijer et al., 2010; Hofmann et al., 2010). Only recently have mindfulness practices been the subject of more in-depth investigation using neuroimaging methods (see SCAN special issue 2013). The current study examined the neural correlates of focused breathing, an exercise that taps one aspect of mindfulness: the ability to focus one’s attention. From this point forward, we will refer to the mindfulness induction called focused breathing (as describe in previous studies, e.g., Arch and Craske, 2006) as a brief mindfulness induction. We examined if this brief mindfulness induction could alter responses to emotional images, measured by event-related potentials (ERPs), relative to a control condition. In addition the current study examined how these ERP responses are related to individual differences in state and trait mindfulness.

Mindfulness is a non-evaluative form of attention; a mental state allowing individuals to maintain full attention to present sensations and ongoing experiences (Marlatt and Kristeller, 1999), while remaining non-judgmental of these experiences. Rather than a relaxation state, mindfulness is mental training that reduces reactive modes of thought, in turn leading to reduced stress and emotional distress (Kabat-Zinn, 1990; Bishop, 2004). In an attempt to operationalize mindfulness, Bishop (2004) outlined the two major components of mindfulness as self-regulation of attention and orientation to experience. Self-regulation of attention involves focusing attention on the present and inhibiting elaborative processing. Orientation to experience involves being oriented to the present moment with an attitude of curiosity, openness, and acceptance. Similarly, Farb et al. (2013) outline several cognitive mechanisms by which mindfulness training leads to these changes, such as decentering of experience (Fresco et al., 2007), a broader context for appraisal (Garland et al., 2011), or “reperceiving” the world (Carmody et al., 2009). Decentering of experience has been highlighted as an overlapping mechanism between cognitive reappraisal and mindfulness (Hayes-Skelton and Graham, 2013). This is not surprising given that decentering involves seeing one’s thoughts and feelings as objective events in one’s mind rather than subjectively experiencing them (Hayes-Skelton and Graham, 2013). Decentering allows one to approach situations non-judgmentally and with the opportunity to interpret the situation objectively.

The neural mechanisms underlying mindfulness are still not well understood. One explanation suggests that long-term mindfulness training alters brain networks involved in interoceptive attention, or attention that is focused on internal states (Farb et al., 2013). Similarly, the attention network is more engaged during a brief mindfulness induction referred to as focused breathing relative to a control condition (Dickenson et al., 2013). Mindfulness training has also been associated with reduced affective Stroop conflict, with those receiving mindfulness training having more dorsolateral prefrontal cortex (DLPFC) activation during conflict resolution (Allen et al., 2012). There is also evidence that mindfulness meditation leads to neuroplastic changes in structures important for self-regulation (for a review see Holzel et al., 2011) and also between particular default mode network regions, likely reflecting enhanced present-moment focus (Taylor et al., 2013). Along the same lines, there is electrophysiological evidence that engaging in regular, brief mindfulness training alters the efficiency of allocating cognitive resources, leading to better self-regulation of attention (Moore et al., 2012). Dispositional mindfulness, how likely a person is to engage in mindful behaviors, is associated with reduced electrophysiological responses to negative images and motivationally salient positive images (erotica) (Brown et al., 2013). Long-term meditators show the same pattern of reduced responses to negative images, but no effect on positive emotional stimuli (Sobolewski et al., 2011). Overall, it appears, in part, that mindfulness practices induce attentional processing changes and heightened recruitment of executive processing and self-regulation resources, likely having implications for how subsequent events are processed.

While previous studies have examined the brain networks and electrophysiological signatures of mindfulness practices, these studies have mostly focused on long-term meditators, self-reported trait mindfulness, or long-term mindfulness training programs. The ability to control attention effectively is important in regulating emotions by decreasing emotionally reactive behaviors (Wenk-Sormaz, 2005; Arch and Craske, 2006) and has implications for processing emotionally salient events. Here we were interested in whether brief bouts of mindfulness, using the focused breathing technique implemented in previous studies that shows particular promise in acutely altering an individual’s attentional resources and state mindfulness, can counter emotional reactivity to negative images.

Event-related potentials are ideal for studying the unfolding of emotional processing, given their excellent temporal resolution. In addition, ERPs can help distinguish the stages of processing during which mindfulness impact processing; dissociating the early attention driven stages of processing and later subjective, evaluative processing. The P300 and late positive potential (LPP) are good candidates for tracking these stages of electrophysiological signatures of emotional processes. Typically the P300 is observed to “oddball” or task relevant stimuli between 300 and 500 ms after presentation of the stimulus. The P300 is one of the most widely studied ERP components and it is sensitive to a wide range of cognitive processes and stimulus characteristics, ranging from probability, task difficulty, resource allocation, and stimulus category to name a few (see Polich, 2011 for a review of the P3; Luck, 2014 for an overview of the P300). For the purposes of this study, we are interested in the P300 response as it relates to processing of emotion stimuli. In studies using emotional stimuli, the P300 has been summarized as reflecting “the allocation of capacity-limited resources toward motivationally salient environment stimuli” and it has been hypothesized that emotional stimuli may act as “natural targets” (Hajcak et al., 2010). Several studies have shown that emotional stimuli automatically capture our attention, producing larger P300’s for emotional images compared to neutral (Radilová, 1982; Johnston et al., 1986; Lifshitz, 1966; Mini et al., 1996). Beyond the P300, a sustained positivity, sensitive to the emotional content of the stimuli, has been observed. This ongoing positivity after the P300, termed the LPP, occurs in an overlapping to later time-window (lasting as long as the stimulus presentation or even longer) and can be difficult to separate from the P300. The LPP has been identified as a component sensitive to the arousal and valence of images, with more arousing positive and negative images producing larger LPPs than neutral images (e.g., Hajcak et al., 2010). As has been shown in studies examining reappraisal, subjective evaluation of the stimulus influences the LPP. The LPP can be decreased by reappraisal during image viewing (Hajcak and Nieuwenhuis, 2006), by presenting a different description of the image (Macnamara et al., 2009), or by directing attention to less arousing aspects of an emotional scene (Dunning and Hajcak, 2009).

While use of explicit, online strategies to regulate one’s emotion are useful, particular strategies (such as attention deployment, reappraisal etc.) may have limited application across a range of experiences one may encounter; therefore, it may be beneficial to adapt a more general frame of mind that influences how attention is deployed to a variety of scenarios such as that encouraged in mindfulness training. Indeed, research examining dispositional mindfulness has found those who are more mindful have smaller LPPs compared to less mindful participants (Brown et al., 2013). While dispositional mindfulness has been examined, the explicit induction of mindfulness in participants while measuring their emotional responses with ERPs has not.

The current study aimed to examine whether a brief mindfulness induction relative to an active control, can alter the amplitude and/or the time-course of electrophysiological indices of emotional processing of images. We were interested in whether a brief induction of mindfulness can provide, in the short-term, the same modulation of emotion sensitive ERP components as longer term mindfulness training (long term meditators) or naturally high trait mindfulness levels. In addition, we were interested in the role of individual differences in the responsiveness to this brief induction.

We predicted that if the brief mindfulness induction condition leads to changes in mindfulness relative to the control condition, participants would have reduced responses (measured by both the P300 and LPP) to emotionally arousing stimuli, specifically to negative compared to neutral images. We did not predict that the brief induction would reduce the P300 or LPP for positive, high arousal images. The P300 reflects attentional salience of the images whereas the LPP reflects subjective evaluation of the image’s motivational significance (Hajcak et al., 2010). Trait mindfulness has been found to modulate the LPP to high arousal, motivationally salient images (erotica) (Brown et al., 2013). In the current study, erotic images are only a small subset of the positive, high arousal images; therefore, we did not expect focused breathing to affect positive image responses. Trait mindfulness has been shown to influence the LPP, therefore we expected, regardless of the condition (brief mindfulness induction or control), there may be differences in the LPP based on individual differences in trait mindfulness, with smaller responses to negative images compared to neutral in those with higher trait mindfulness. Alternatively, an individual’s state mindfulness may also influence these components that reflect emotional processing.

## Materials and Methods

### Participants

Twenty-four participants (13 males, 18–22 years old, *M* = 20.4, *SD* = 1.2 years) participated in this study for monetary compensation. Participants had normal or corrected-to-normal vision and had no history of neurological or psychiatric illness. In addition, sub-clinical high levels of anxiety and depression were screened for with the Hospital Anxiety and Depression Scale (HADS), and any participant with a score of 11 or higher was excluded from the study. Participants gave informed consent in accordance with the Tufts University Institutional Review Board and the Army Human Research Protections Office.

### Materials

Three-hundred and sixty images were taken from the International Affective Picture System (IAPS; Lang et al., 2008). The subset of images used in the current experiment consisted of 120 neutral, moderate arousal (*M_valence_* = 4.61, *SD_valence_* = 0.3; *M_arousal_* = 3.67, *SD_arousal_* = 0.9); 120 negative valence, high arousal (*M_valence_* = 2.42, *SD_valence_* = 0.36; *M_arousal_* = 5.88, *SD_arousal_* = 0.5); and 120 positive valence, high arousal images (*M_valence_* = 7.19, *SD_valence_* = 0.5; *M_arousal_* = 5.8, *SD_arousal_* = 0.6). The mean and SD reported here reflect the 1–9 self-assessment manikin values used to evaluate the IAPS (see Lang et al., 2008 for details on the normative ratings). The positive and negative images did not differ on arousal from one another [*t*(238) = 0.97, *p* = 0.334], but did differ significantly from the neutral images on arousal [neutral vs. negative: *t*(238) = 22.52, *p* < 0.001; neutral vs. positive: *t*(238) = 20.9, *p* <0.001]. All three conditions differed significantly on valence [*F*(2,359) = 4813.45, *p* < 0.001]. The stimuli were divided into two lists (60 of each condition) that did not differ on arousal between the negative and positive images. Across sessions, each images was seen an equal number of times.

#### Individual Differences Measures

To account for potential differences in emotional reactivity to the stimuli between participants based on susceptibility to and acceptance of mindfulness-based practices, we administered two trait mindfulness scales [Mindful Attention Awareness Scale (MAAS), and the Five Facet Mindfulness Questionnaire (FFMQ)] at the first visit. At each visit, self-rated mood state [International Positive and Negative Affect Schedule Short Form (I-PANAS-SF)], and state mindfulness [Toronto Mindfulness Scale (TMS)] were collected and used as dependent measures in addition to ERPs.

##### Mindful Attention Awareness Scale

The MAAS is a 15-item questionnaire assessing trait aspects of mindfulness. The items are rated on a scale from 1(almost always) to 6(almost never). The total score is computed by taking the average of the responses to the 15 items. Higher scores reflect higher levels of dispositional mindfulness.

##### Five Facet Mindfulness Questionnaire

The FFMQ is a 39-item questionnaire also assessing trait mindfulness on five subscales: Observe (eight items) higher scores = more observant (highest possible score = 40), Describe (eight items) higher scores = more descriptive (highest possible score = 40), Act with Awareness (eight items) higher scores = more aware of actions (highest possible score = 40), Non-judge (eight items) higher scores = less judgmental (highest possible score = 40) and Non-react (seven items) higher score = better able to not react (highest possible score = 35). Each item is rated on a scale from 1(never or rarely true) to 5(very often or always true). Each subscale score is computed by summing the ratings for each item.

##### Toronto Mindfulness Scale

The TMS is a 13-item questionnaire measuring a heightened focus of attention to internal states and to a lesser degree one’s environment. The items are rated on a scale of 0(not at all) to 4(very much). Two sub-scales are computed: curiosity and decentering. The curiosity subscale reflects an attitude of wanting to learn more about one’s experiences and the decentering subscale reflects a shift from identifying personally with thoughts and feelings to relating to one’s experience in a wider field of awareness. Each subscale score is calculated by summing the responses to each question. The maximum score on the curiosity scale is 24, and the highest score on the decentering scale is 28. Higher scores reflect higher state mindfulness.

##### International Positive and Negative Affect Schedule Short Form

This 10-item questionnaire consists of five items assessing positive emotional states and five items assessing negative emotional states. Each item is scored on a scale of 1 = not at all to 5 = extremely. The maximum score on each scale (positive and negative) is 25. Higher scores equal higher intensity of emotion. For the current study we computed difference scores by subtracting the baseline measure from the other two taken after the breathing exercise and after IAPS viewing.

#### Brief Mindfulness Induction and Control Conditions

These conditions were created from Kabat-Zinn’s (2005) Guided Mindfulness Meditation practice CDs (disk 3, *Sitting Meditation*). Two exercises were developed for use in this experiment and were modeled after Arch and Craske’s (2006) experiment design. For both the brief mindfulness induction and control conditions, participants listened to a 15-min recording instructing them to establish a straight upright sitting posture, hands resting on their lap, shoulders relaxed, head upright, and feet resting flat on the floor. If comfortable doing so, they were asked to close their eyes; if not, they were asked to direct gaze slightly downward and forward without focusing on anything in particular. For the *brief mindfulness induction condition*, participants were guided through instructions and engaged in the practice of focusing on the sensations of inhalation and exhalation. In addition they were instructed to be fully present in the moment, to bring their attention back to the sensation of breathing when their mind wanders. In addition, the concept of mindfulness is emphasized by encouraging the participant to cultivate an attitude of gentleness and being non-judgmental when their mind wanders from attending to the sensation of breathing. For the *control condition*, participants were repeatedly instructed to “simply think about whatever comes to mind. Let your mind wander freely without trying to focus on anything in particular.” Both recordings lasted 15 min, and the brief mindfulness induction condition has been shown to reliably increase attentional control and aid in regulating negative affect (Brunyé et al., 2013).

### Procedure

Participants came into the laboratory for two sessions and either took part in the brief mindfulness induction condition or the control condition (the order was counterbalanced across participants). On the first visit, after obtaining informed consent, participants filled out trait mindfulness questionnaires (MAAS, FFMQ), and anxiety and depression questionnaire (HADS), in addition to self-rated mood (I-PANAS-SF). Prior to engaging in the focused breathing/unfocused control, participants were fitted with an EEG cap. Participants were randomly assigned (order counterbalanced across participants) to one of two exercises for the first visit and the other for the second visit. Participants listened to the recording (either the brief mindfulness induction or control depending on the session) for 15 min in a quiet room with the door closed.

After completing the exercise, participants completed the I-PANAS-SF again and then passively viewed IAPs images for approximately 15 min. Participants viewed 180 items total; 60 neutral, 60 negative, and 60 positive images. The images were presented in pseudo-random order, each for 1500 ms with an inter-stimulus interval of 500 ms. Images were presented at 1024 × 768 on a 19-inch CRT monitor at a viewing distance of 120 cm. They were instructed to naturally experience the images as they viewed them. After viewing the IAPs images, participants completed the I-PANAS-SF a final time as well as the TMS.

**FIGURE 1:**
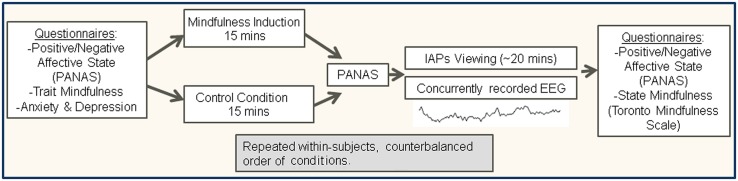
**Overview of experimental procedure**.

At least 1 week later, participants returned for the second session where the procedure was identical to the first visit except for the recording they listened to; if they had completed the brief mindfulness induction at the first session, they did the control condition at the second session and vice-versa (note they did not complete MAAS, FFMQ, or HADS again – see **Figure 1** for study procedure).

### EEG Recording

**FIGURE 2:**
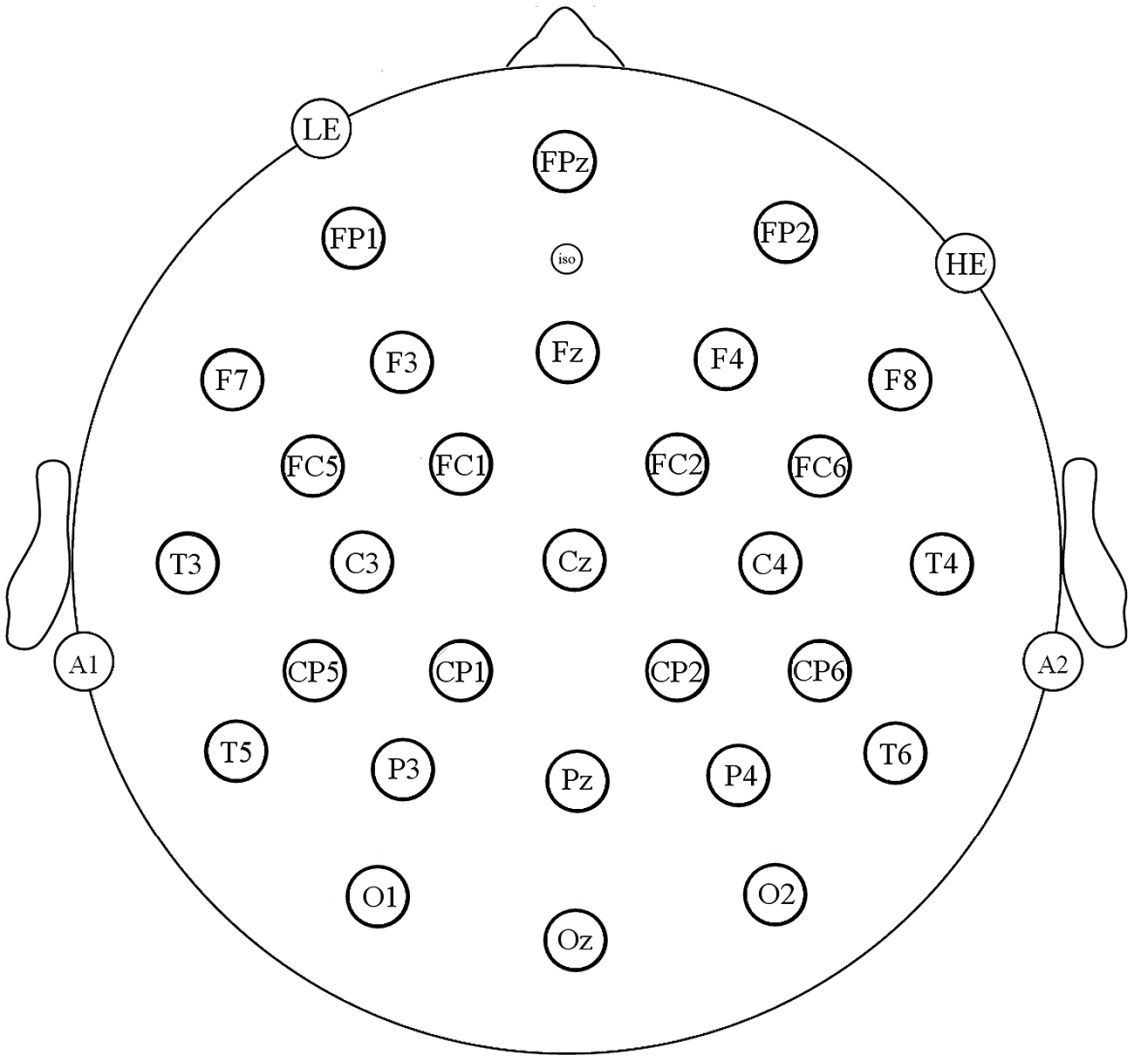
**Recording montage**.

Prior to engaging in the brief mindfulness induction or control condition exercise, participants were fitted with a 29-channel electrode cap (Electro-cap International) to collect the electroencephalogram (EEG; see **Figure 2** for electrode locations). In addition, three external electrodes were placed to monitor vertical and horizontal eye activity and differential mastoid activity. All electrodes, including one over the right mastoid, were referenced to an electrode over the left mastoid (oﬄine all channels were referenced to an average of the mastoids). Horizontal and vertical eye movements and blinks were detected from electrodes placed below and to the side of the eyes, and scalp impedances were lowered to below 10 kΩ. The EEG (250 Hz sampling rate, bandpass 0.01 and 40 Hz) was recorded continuously with an SA Instruments amplifier (San Diego, CA, USA).

### Data Analysis

#### Questionnaire Scores

The trait mindfulness scores (MAAS, FFMQ) cannot be compared between the two conditions since they are trait measures; however, later in the analysis these scores are correlated with ERP effects. Scores on the TMS scale were compared between conditions (brief mindfulness induction vs. control) using a paired samples *t*-test. Scores on the I-PANAS-SF positive and negative scales were separately entered into a repeated measures ANOVA with the factors of measurement time (after the brief mindfulness induction or control exercise minus baseline, and after viewing the IAPS minus baseline) and condition (brief mindfulness induction, control). Main effects were followed up with Bonferonni corrected *t*-tests. In addition to this repeated measures ANOVA a paired *t*-test was performed to ensure participants did not differ in negative and positive affect at the start of the session between the two conditions. To perform a manipulation check we also compared the baseline measurement to the second measurement (after brief mindfulness induction, after control) for each session separately on the negative affect scale as we expected the brief mindfulness exercise to reduce negative affect and did not expect the control condition to have an effect.

#### ERP Data

For each session, ERP averages were formed by time-locking to the onset of the images for each valence and condition separately from 200 ms prior to image onset until 1500 ms after. Trials with blinks, eye movements, and muscle artifact were rejected prior to averaging (∼20% of all trials). The number of trials per condition did not differ significantly between sessions or conditions (paired *t*-tests, *p*’s > 0.3; mean number of trials (out of 60) per condition (brief mindfulness condition: *M_negative_* = 48.5, *SD_negative_* = 5.7, *M_neutral_* = 47.6, *SD_neutral_* = 5.3, *M_positive_* = 48.4, *SD_negative_* = 6.0; control condition: *M_negative_* = 47.1, *SD_negative_* = 6.0, *M_neutral_* = 48.6, *SD_neutral_* = 4.6, *M_positive_* = 47.2, *SD_negative_* = 5.3). Mean amplitude measurements were taken in three time windows to quantify the P300 (300–500 ms) and the LPP (500–1500 ms). The two time windows were based on previous studies that have quantified the P300 in this time window (Lifshitz, 1966; Radilová, 1982; Johnston et al., 1986; Mini et al., 1996) and the LPP was characterized as the remaining picture viewing time (500–1500 ms). A centro-parietal region was computed by averaging across electrodes CP1, CP2, P3, Pz, and P4 as the P300 and LPP are typically observed in centro-parietal electrodes (Hajcak et al., 2010; Polich, 2011). Prior studies examining the LPP have created a similar region of interest using centro-parietal and parietal electrodes (Dunning and Hajcak, 2009; Hajcak et al., 2013)^1^. Repeated measures ANOVA’s were performed with these mean amplitudes including the within-subjects factors of condition (brief mindfulness induction, control) and valence (negative, positive, neutral). Planned comparisons using uncorrected *t*-tests (two-tailed) were performed on the difference wave values for negative minus neutral and positive minus neutral to compare the brief mindfulness induction and control conditions as we hypothesized that the two conditions may differ specifically on emotional processing on the P300 and LPP. For all other *post hoc* comparisons, the Bonferroni correction was applied and the adjusted critical *p* values are reported. Partial eta square ($\eta_{p}^{2}$) and Cohen’s *d* are reported to quantify effect size.

#### Relating Individual Differences to ERP Effects

Because we were interested in how individual differences in state and trait mindfulness related to ERP effects, we correlated mean amplitude of the difference waves (negative minus neutral; positive minus neutral) for each of the conditions (brief mindfulness induction, control), in the two time epoch analyzed with state and trait mindfulness measures. These mean amplitude difference wave values were computed from the centro-parietal region average. For the TMS, we correlated each subscale (decentering, curiosity as well as the total score) separately with mean amplitude difference waves. For trait mindfulness we correlated the MAAS and FFMQ the overall scores were with mean amplitude difference waves.

## Results

### Questionnaire Scores

See **Table 1** for a summary of trait mindfulness scores (MAAS, FFMQ) and **Table 2** for state mindfulness score (TMS) and I-PANAS-SF ratings for the brief mindfulness induction and control conditions.

**Table 1:** Means and standard deviations for MAAS and FFMQ (Trait Mindfulness).

Measure	*M*	*SD*
MAAS	4.45	0.68
FFMQ – Total	28.40	2.90

**Table 2:** Means and standard deviations for TMS and I-PANAS-SF.

	Mindfulness condition		Control condition	
Measure	*M*	*SD*	*M*	*SD*
TMS curiosity	15.58	4.82	15.38	4.16
TMS decentering	17.58	4.04	16.5	4.02
PANAS positive score after exercise	-2.46	3.25	-2.00	2.57
PANAS negative score after exercise	-0.92	1.95	-0.25	1.19
PANAS positive score after IAPS	-1.38	2.43	-1.17	2.44
PANAS negative score after IAPS	0.23	1.35	0.29	1.55

*Scores are difference from baseline (e.g., score after exercise – baseline), negative scores reflect a decrease in mood from baseline.*

#### Toronto Mindfulness Scale

When comparing state mindfulness scores between the brief mindfulness induction and control conditions, there were no significant differences between TMS scores (decentering and curiosity subscales) between the two conditions (all *p*’s > 0.23, see **Table 2** for means and *SD*s).

#### International Positive and Negative Affect Schedule Short Form

**Table 2** summarizes the positive and negative ratings from the I-PANAS-SF for the brief mindfulness induction and control conditions at two different time points relative to baseline (after breathing – baseline and after IAPS viewing – baseline). Examining I-PANAS-SF ratings at the start of the session, ratings on the negative and positive scales did not differ between the brief mindfulness induction and control conditions (*p*’s > 0.5). To examine if the brief mindfulness induction and control conditions differed at any other point during the experiment we performed a two condition (brief mindfulness induction, control) × 2 time (after exercise; after IAPS) repeated measures ANOVA for each rating subscale (positive/negative) separately. For the negative subscale, there was a main effect of time, *F*(1,23) = 10.77, *p* = 0.003, $\eta_{p}^{2}$ = 0.32, as well as a trend toward time and condition interacting, *F*(1,23) = 3.51, *p* = 0.074, $\eta_{p}^{2}$ = 0.13. The main effect of time reflects more negative ratings from baseline when measuring negative affect after the brief mindfulness induction condition and a reduction in negative affect at the end of the experiment. The trending interaction reflects less negative ratings in the brief mindfulness induction condition compared to control after the exercise and similar ratings at the end of the experiment (brief mindfulness induction condition, after exercise - baseline: *M* = -0.917, *SD* = 1.95; brief mindfulness induction condition, end of experiment – baseline: *M* = 0.229, *SD* = 1.35; control condition, after exercise – baseline: *M* = -0.25, *SD* = 1.19; control condition, end of experiment – baseline: *M* = 0.292, *SD* = 1.55). However, this effect did not reach significance, *t*(23) = 1.62, *p* = 0.119, *d* = 0.39. On the positive scale, there was also a main effect of time, *F*(1,23) = 7.34, *p* = 0.013, $\eta_{p}^{2}$ = 0.24, with scores becoming more positive toward the end of the experiment (after exercise – baseline: *M* = -2.23, *SD* = 2.46; end of experiment – baseline: *M* = -1.27, *SD* = 1.85. There was no main effect of condition or interaction with condition (*F*’s<0.32, *p*’s > 0.5).

As a manipulation check we compared negative affect scale ratings at baseline to after the brief mindfulness induction exercise and ratings baseline to after the control condition separately. For the brief mindfulness induction condition, negative affect decreased after the exercise relative to baseline, *t*(23) = 2.30, *p* = 0.031, *d* = 0.35, *M_baseline_* = 6.63, *SD_baseline_* = 3.06, *M_afterbreathing_* = 5.71, *SD_afterbreathing_* = 2.03. However, for the control condition, negative affect did not change from baseline to after the control exercise, *t*(23) = 3.81, *p* = 0.314, *d* = 0.07, *M_baseline_* = 6.83, *SD_baseline_* = 3.46, *M_after__control_* = 6.58, *SD_after__control_* = 3.53. However, as noted above, the difference from baseline to after breathing exercise did not differ significantly between the two sessions (*p* = 0.119).

### ERP Results

**FIGURE 3:**
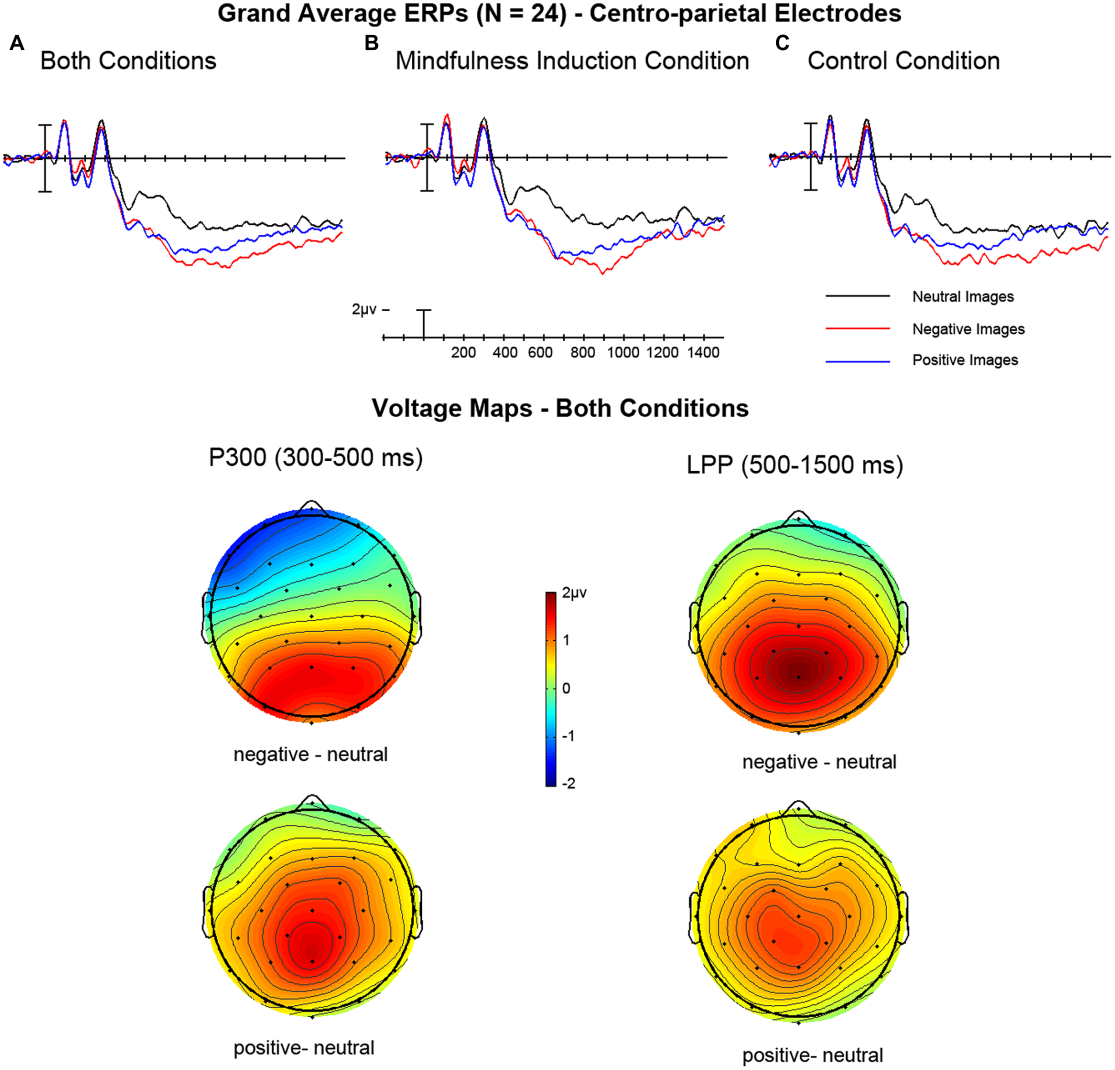
**(Top)** Grand average ERPs showing the average across centro-parietal electrodes (CP1, CP2, P3, Pz, P4). (A) ERPs for neutral, negative, and positive images collapsed across the brief mindfulness induction and control conditions. (B) ERPs for the brief mindfulness induction condition. (C) ERP for the control condition. **(Bottom)** Voltage maps for P300 and LPP collapsed across brief mindfulness induction and control conditions to demonstrate the distribution of the P300 and LPP for negative minus neutral and positive minus neutral images.

**Figure 3** shows the grand average ERPs and voltage distributions for 24 participants for the two sessions, brief mindfulness induction and control, as well as collapsed across condition. **Table 3** summarizes the mean amplitudes for each of the conditions by image valence. To ensure session order did not affect the ERPs of interest, we included session order as a between-subjects factor in the ANOVA’s. We did not find any main effects or interactions of session order (all *p*’s > 0.27). This factor was not included in subsequent ANOVA’s. Below we outline the findings for the two ERP effects of interest, the P300 and the LPP.

**Table 3:** Means and standard deviations of ERP mean amplitudes (μV).

Time window	Image type	Mindfulness condition		Control condition	
		*M*	*SD*	*M*	*SD*
P300 300–500 ms	Negative	2.00 μV	5.59 μV	2.86 μV	5.62 μV
	Neutral	1.08 μV	4.97 μV	1.67 μV	4.99 μV
	Positive	2.29 μV	5.21 μV	3.03 μV	5.35 μV
LPP 500–1500 ms	Negative	5.82 μV	2.69 μV	6.30 μV	2.90 μV
	Neutral	4.15 μV	2.22 μV	4.86 μV	2.80 μV
	Positive	5.41 μV	2.03 μV	5.47 μV	2.59 μV

#### P300 (300–500 ms) Epoch

The overall within-subjects repeated measures ANOVA compared condition (focused, unfocused) and valence (negative, neutral, positive) within the centro-parietal electrodes. This analysis showed a main effect of condition with the control condition having a slightly more positive-going deflection than the brief mindfulness induction condition, *F*(1,23) = 4.81, *p* = 0.039, $\eta_{p}^{2}$ = 0.17, see **Table 3** for means and *SD*s. There was also main effect of valence, *F*(2,46) = 12.13, *p* < 0.0001, $\eta_{p}^{2}$ = 0.35, driven by both negative and positive images having more positive going amplitudes than neutral images (*p*’s < 0.002, see **Table 3** for means and *SD*s), but not differing from one another (*p* = 0.34, Bonferroni adjusted critical *p* = 0.0167). There was no interaction between valence and condition, *F*(2,46) = 0.2, *p* = 0.82, $\eta_{p}^{2}$ = 0.01. Because we were specifically interested in how the brief mindfulness induction may impact affective processing, *a priori* paired *t*-tests were performed for the mean amplitude difference between negative and neutral images and positive and neutral images to compare the two conditions (brief mindfulness induction vs. control). No difference between the brief mindfulness induction vs. control conditions was found for the effect of negative images, *t*(23) = 0.65, *p* = 0.520, *d* = 0.15, or positive images, *t*(23) = 0.40, *p* = 0.696, *d* = 0.1.

#### LPP (500-1500 ms) Epoch

The ANOVA comparing condition (brief mindfulness induction, control) and valence (negative, neutral, positive) within the centro-parietal electrodes revealed a significant main effect of valence, *F*(2,46) = 18.21, *p* < 0.00001, $\eta_{p}^{2}$ = 0.44. There was no significant main effect of condition, *F*(1,23) = 2.55, *p* = 0.12, $\eta_{p}^{2}$ = 0.1, and the interaction between condition and valence was not significant, *F*(2,46) = 1.88, *p* = 0.16, $\eta_{p}^{2}$ = 0.1. The main effect of valence was followed-up with corrected comparisons, which revealed this effect was driven by both negative and positive images having more positive going amplitudes than neutral images (*p*’s < 0.001), but not differing from one another (*p* = 0.030, Bonferroni adjusted critical *p* = 0.0167, see **Table 3** for means and *SD*s). Because the condition × valence interaction did not reach significance, follow-up comparisons were not performed. However, planned comparisons between the brief mindfulness induction and control conditions on the negative minus neutral and positive minus neutral difference revealed a trend toward a difference in processing of positive images, *t*(23) = 1.32, *p* = 0.054, *d* = 0.48, but not negative images, *t*(23) = 1.01, *p* = 0.529, *d* = 0.14. For the positive versus neutral comparison, the brief mindfulness induction condition (*M_positive-neutral_* = 1.27 μV) had a greater positive-going LPP than the control condition (*M_positive-neutral_* = 0.61 μV).

### Individual Differences and ERP Effects

**FIGURE 4:**
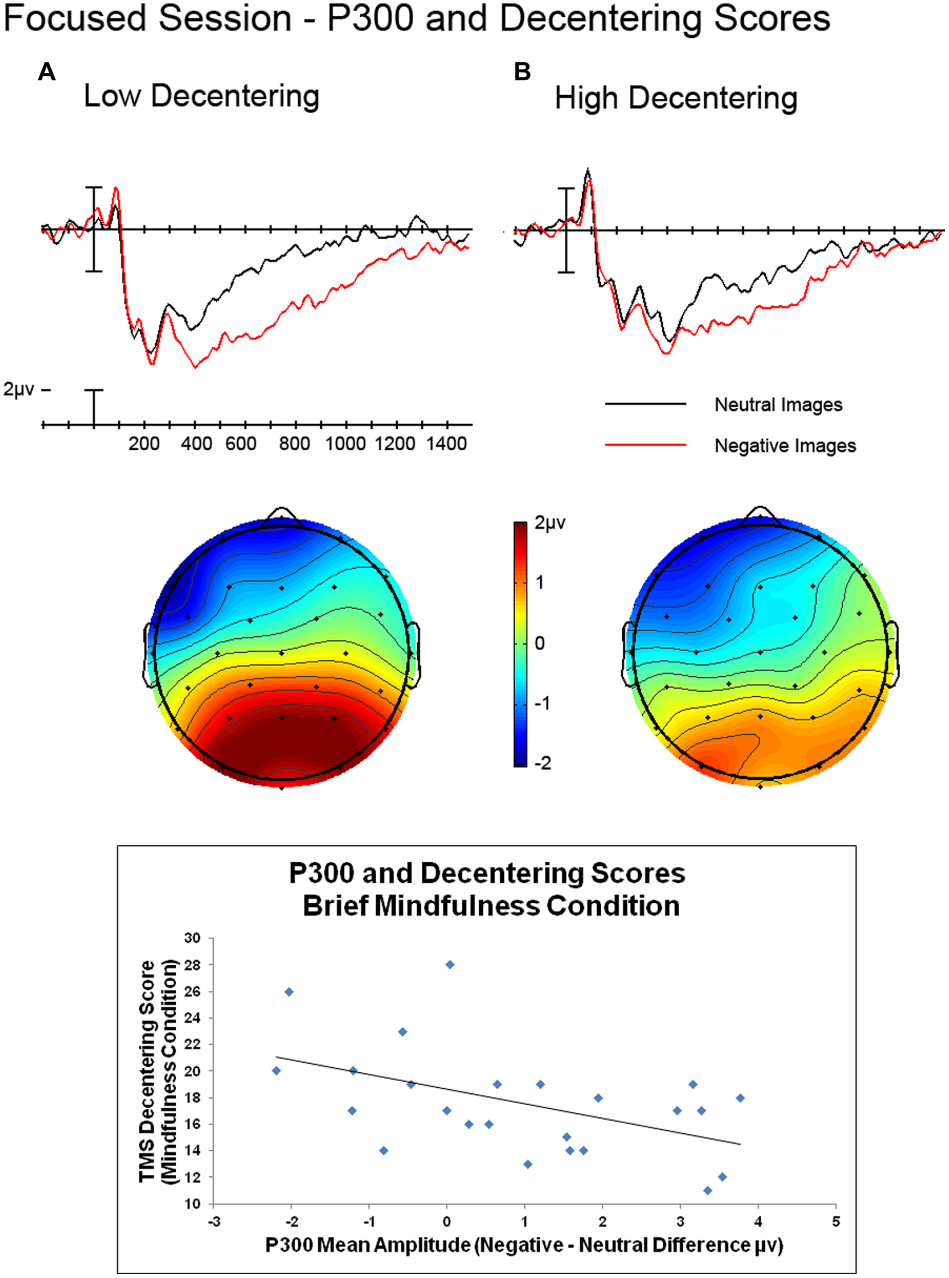
**Grand average ERPs comparing negative and neutral images for median split based on TMS Decentering Scores for the brief mindfulness induction condition. (Top)** Waveforms plotted are from the average of the centro-parietal electrodes. Voltage maps reflect the difference values of negative – neutral mean amplitude during the P300 epoch (300–500 ms). **(Bottom)** Scatterplot of decentering scores and mean amplitude difference negative and neutral images from the average of the centro-parietal electrodes during the P300 epoch.

We expected that individuals’ abilities to engage effectively in the brief mindfulness induction may influence the pattern of ERP effects we observed in the current study. Therefore, we examined the relationship between state and trait mindfulness and ERP effects in the time epochs analyzed above (see **Tables 4** and **5** for a summary of the correlational results). In the P300 epoch, we found that state mindfulness, specifically Decentering on the TMS, was related to the size of the difference between negative and neutral images for the brief mindfulness induction condition. The higher the decentering score, the smaller the P300 response to negative images relative to neutral, see **Figure 4**, *r*(24) = 0.494, *p* = 0.014.

**Table 4:** Pearson correlation coefficients for trait mindfulness scores and ERP effects.

	P300			
	Mindfulness condition		Control condition	
Measure	Negative – Neutral	Positive – Neutral	Negative – Neutral	Positive – Neutral
MAAS	0.321	0.282	0.075	0.104
FFMQ	0.219	0.264	0.209	0.337

	**LPP**			
	**Mindfulness condition**		**Control condition**	
	**Negative – Neutral**	**Positive – Neutral**	**Negative – Neutral**	**Positive – Neutral**

MAAS	0.385	0.433^∗^	0.027	0.123
FFMQ	0.165	0.329	-0.053	-0.121

*^∗^p < 0.05.*

**Table 5:** Pearson correlation coefficients for state mindfulness scores on TMS and ERP effects.

	P300			
	Mindfulness condition		Control condition	
Measure	Negative – Neutral	Positive – Neutral	Negative – Neutral	Positive – Neutral
Total	-0.215	-0.116	-0.253	0.185
Decentering	-0.494^∗^	-0.296	-0.103	0.322
Curiosity	0.105	0.082	-0.259	-0.049

	**LPP**			
	**Mindfulness condition**		**Control condition**	
	**Negative – Neutral**	**Positive – Neutral**	**Negative – Neutral**	**Positive – Neutral**

Total	-0.012	0.032	0.058	-0.114
Decentering	-0.331	-0.117	-0.062	0.226
Curiosity	0.261	0.144	-0.102	0.036

*^∗^p < 0.05.*

In the later time epochs, reflecting the LPP, MAAS scores were positively related to the LPP amplitude when comparing positive and neutral images. Participants with higher trait mindfulness scores on MAAS had larger LPPs difference between positive and neutral images in the brief mindfulness induction condition, MAAS: *r*(24) = 0.433, *p* = 0.034^2^.

## Discussion

In the current study we found that a brief mindfulness induction relative to an active control, did not result in reduced ERP indices of emotional processing as measured by the P300 and LPP. We did observe a trend toward a larger LPP for processing positive stimuli in the brief mindfulness induction condition compared to the control condition. Interestingly, however, an individual’s ability to effectively engage in the brief mindfulness induction as measured by state mindfulness, specifically, the ability to maintain a decentered state during emotional image viewing, was related to reduced ERP responses to emotional images when comparing negative images to neutral images in the P300 epoch. While an effect of the brief mindfulness induction compared to the control condition did not produce ERP differences to processing of emotion images, an effect on processing of negative images was only observed when taking into account participants’ self-reported state mindfulness after imaging viewing.

While the data showed trends toward differences between the two conditions, the direction indicated *increased* responses to emotional images for the brief mindfulness induction condition. This pattern was not of the predicted direction, but it is not surprising given that participants reported feeling more alert and attentive after engaging in the brief mindfulness induction relative to the control condition. This more attentive state led to increased, attentive processing of these images. Participants who not only were more alert, but also were more mindful in how they focused their attention, were able to better regulate their response to the negative images. *Decentering* is defined as the ability to objectively evaluate a situation, knowing that while you are fully aware of your environment, you can choose whether or not to allow what is happening to influence your emotional state. The fact that state mindfulness, reflected by the decentering score on the TMS, influenced emotional processing during the P300 epoch suggests that the change in mindfulness affects processing prior to the subjective evaluation of the stimulus reflected by the LPP and instead reflects the allocation of attention to these stimuli. This finding is in line with the idea that focused attention, induced by mindfulness training and focused breathing exercises, leads to better attentional control (Kozasa et al., 2012), an increase in interoceptive attention (Farb et al., 2013), and engages the fronto-parietal attention network (Dickenson et al., 2013).

We did not replicate a reduction in LPP amplitude to negative images related to trait mindfulness, as measured by the MAAS (Brown et al., 2013). Instead we found that participants with higher trait mindfulness scores on the MAAS had larger LPP effects for positive images compared to neutral images. One reason for failing to replicate this finding could be the method by which we defined the LPP for negative images. We calculated the mean amplitude difference between negative and neutral images in the LPP time epoch, which we term the “LPP effect,” whereas, Brown et al. (2013) used the amplitude of the negative image condition without comparison to another condition. However, this seems unlikely given that correlations with negative image amplitude only in our two LPP epochs still did not show this same pattern. Another reason for the discrepant findings could be that the state induction of mindfulness may have negated trait mindfulness effects. As previously mentioned, there was a trend toward larger LPP effects during the brief mindfulness induction compared to the control condition. The attentional focus induced by the brief mindfulness induction may have temporarily negated the beneficial effects of trait mindfulness.

We found no correlation between our state mindfulness measures and trait mindfulness measured by the MAAS (all *r’s* < 0.2). This suggests that while some participants may have been more mindful than others, this did not influence their state mindfulness after practicing focused breathing. One limitation of the current study is that state mindfulness was measured at the end of the experiment rather than after the brief mindfulness induction/control condition exercises and therefore the state mindfulness measure was more likely influenced by IAPs viewing rather than the mindfulness exercise. In addition, when examining ERP responses during the brief mindfulness induction condition, we only found reduced responses on the P300 component when taking into account participants’ ability to adopt a decentered perspective. Brown et al. (2013) found their effects during a later epoch suggesting different mechanisms operating between our experiment and theirs. In the current experiment focused breathing led to differences in attentional allocation to emotional stimuli, as reflected by the P300, whereas in their study, trait mindfulness led to effects on the LPP, reflecting subjective evaluation of the stimuli.

Another limitation of the current study is the relatively small sample size. It is possible some of the null effects found in this study were due to insufficient power. A larger sample size would allow for a better characterization of the relationship between the ERP effects and individual differences measured in this study.

While this study did not find overall effects of the brief mindfulness induction, mindfulness practices hold promise for modulating emotional responses to stimuli. This study highlights the importance of an individual’s ability to engage in the mindfulness practice. One possibility is that if participants took part in multiple or longer sessions more robust effects of the mindfulness induction might be observed. For example, one study found that 3 days of mindfulness mediation for 20 min a day led to significant decreases in pain ratings (Zeidan et al., 2010b) and another found better sustained attention after 4 days of mindfulness meditation training (Zeidan et al., 2010a,b). However, other studies have provided inconclusive evidence arguing for a particular length of mindfulness training to observe effects (for a review Carmody and Baer, 2009). In addition, the brief mindfulness induction focused on a several aspects of mindfulness, focusing attention on one’s breathing and avoiding mind-wandering in addition to cultivating non-judgmental attitudes. In order to better understand the exact mechanisms underlying the mindfulness manipulation in this study, control conditions could be implemented in future studies that control for the different instructions given. One control condition for comparison could focus on attention to breath solely and another could focus on cultivating a non-judgmental attitude.

However, this study demonstrates that ERPs to emotional stimuli can differentiate state influences of mindfulness after a brief mindfulness induction. Previous studies have mainly focused on how mindfulness modulates responses to negative emotional stimuli; our findings suggest that mindfulness inductions may also have an effect on processing of positive stimuli, where a trend toward an increase in LPP, likely reflecting subjective evaluation of positive images, was observed after the brief mindfulness induction. Future studies should also focus on how mindfulness modulates responses to positive stimuli and how enhanced processing of positive stimuli may also prove to be a beneficial outcome of mindfulness practices.

## Conflict of Interest Statement

The authors declare that the research was conducted in the absence of any commercial or financial relationships that could be construed as a potential conflict of interest.

## Footnotes

1 The analysis was also conducted including frontal, central, and parietal (F3, Fz, F4, C3, Cz, C4, P3, Pz, P4) electrodes with the factors of anterior–posterior and hemisphere. This analysis produced a comparable pattern of findings, therefore, for simplicity, the analysis is reported using the collapsed centro-parietal region.

2 Upon examining the waveforms for the low and high decentering groups, it appears there is an effect occurring prior to the P300, in the 200–300 time window. We ran a correlational analysis and found no relationship between the mean amplitude difference between negative and neutral images and TMS Decentering Scores for the brief mindfulness induction condition.
